# Miner’s Physicians’ Memorandum Book

**Published:** 1884-12

**Authors:** 


MINER'S PHYSICIAN'S MEMORANDUM BOOK. A New Weekly Visiting List with Clinical Columns and Ledger Sheets. (31 or 62 patients.) Fifth Improved Edition.

CONTENTS.—Calendar; List of medicines and Doses; Doses for Children; Drops to a drachm; Obstetric Calendar; Metric Tables; Poisons—Symptoms and Treatment (very full); List of Diseases and the best recommended medicines in their treatment; Urinary Analysis and its clinical significance; Hints in Emergencies; Weekly Record Sheets; Ledger Sheets; Obstetric Record; Death Record; Appointments and addresses; Cash Account; Monthly Memoranda; Erasable Tablet.

